# Fish community responses to restoration of a eutrophic coastal bay

**DOI:** 10.1007/s13280-023-01907-3

**Published:** 2023-08-05

**Authors:** Lena Bergström, Ronny Fredriksson, Ulf Bergström, Emil Rydin, Linda Kumblad

**Affiliations:** 1 Department of Aquatic Resources, Swedish University of Agricultural Sciences, Box 7018, 750 07 Uppsala, Sweden; 2 Stockholm University Baltic Sea Centre, 106 91 Stockholm, Sweden

**Keywords:** Baltic Sea, Björnöfjärden, Eutrophication, Fish, Measures, Nutrient reduction

## Abstract

Interest in coastal restoration measures is increasing, but information about subsequent ecosystem recovery processes is limited. In Björnöfjärden on the Baltic Sea coast, Stockholm archipelago, a pioneering case study to reduce coastal eutrophication led to improvements and initially halved phosphorus levels. Here, we evaluate the effects of the restoration on the local fish assemblage over one decade after the measures. The study gives a unique possibility to evaluate responses of coastal fish to nutrient variables and abatement in a controlled natural setting. Cyprinid abundance decreased and perch partially increased with decreasing turbidity levels, while mean trophic level increased over time in the restored area. Responses were overall weak, likely attributed to an attenuation of the eutrophication abatement effect over time. The results suggest that nutrient reduction gives slow responses in fish compared to alternative measures such as fishing closures.

**Supplementary Information:**

The online version contains supplementary material available at 10.1007/s13280-023-01907-3.

## Introduction

Aquatic environments are continuously being degraded due to impacts from human activities (de Groot et al. 2012; Korpinen et al. 2021), and along with the need to reduce pressure levels, the role of active measures to recover biodiversity and ecosystem services is increasingly recognized (Duarte et al. 2020; EC 2020). The UN Decade on Ecosystem Restoration (2021–2030) underscores the urgency of reviving damaged ecosystems. So far, however, there is limited empirical knowledge about the effectiveness of restoration measures and subsequent ecosystem recovery processes in coastal and marine areas (Bayraktarov et al. 2016, 2020), and there is a need for quickly improving the evidence base to enable cost-efficient large-scale restoration efforts (Cooke et al. 2019).

In the brackish Baltic Sea, a semi-enclosed sea in northern Europe, human-induced eutrophication is a prevalent long-lasting pressure, requiring vast efforts to promote recovery of the ecosystem. Excessive anthropogenic loading of nitrogen and phosphorus since the mid-1900's has caused widely distributed eutrophication symptoms (Gustafsson et al. 2012; Bonsdorff 2021), including increased turbidity, changes in species composition and oxygen deficiency, also affecting the provisioning of ecosystem services (Bonsdorff et al. 1997; Carstensen et al. 2014; HELCOM 2018a). Today, regionally coordinated measures have led to significantly decreased loading. However, inputs are still too high (Saraiva et al. 2019; Markus Meier et al. 2021). Further, since nutrients have built up over time and because of the relatively slow rate of water exchange in the Baltic Sea, it is expected to take decades for the Baltic Sea to fully recover naturally to good eutrophication status, even after nutrient inputs have reached target levels (HELCOM 2023). A key problem is that phosphorus is accumulated in internal nutrient pools, which leads to self-sustained eutrophication due to phosphorus leakage under hypoxic conditions (Vahtera et al. 2007). Anoxic and hypoxic areas are mainly widespread in deeper parts of the Baltic Sea, with negative effects on for example benthic communities and species dependent on these for food (Conley et al. 2009; Ehrnsten et al. 2020; Orio et al. 2021). Eutrophication-associated deoxygenation is however also present in coastal areas, particularly in archipelagos and enclosed basins where organic matter accumulates through sedimentation (Bonsdorff et al. 1997; Conley et al. 2011; Puttonen et al. 2014; Carstensen et al. 2020). Estimations have given that 86% of coastal waters in the Baltic Sea region (by area) suffer from eutrophication to some extent, as documented by effects in the water column (e.g., enhanced nutrient concentrations, reduced water transparency, increased algal blooms), benthic habitat (e.g., hypoxia), or species (e.g., altered species composition or reduced depth distribution of plants) (HELCOM 2018a).

Although fish are not expected to respond directly to changes in nutrient levels, eutrophication can affect them through indirect pathways. Fish recruitment and productivity can be affected if eutrophication alters the quality of spawning and nursery habitats, or food availability (Bergström et al. 2013; Hansen and Snickars 2014; Jokinen et al. 2016; Orio et al. 2021). In addition, poor oxygen conditions may affect fish physiology, and changes in water transparency can influence foraging efficiency, with effects on for example spatial distribution or growth (Sandström and Karås 2002; Limburg and Casini 2018). In the Baltic Sea, long-term changes in coastal fish communities have partially been attributed to species tolerant of eutrophication effects being benefitted over more sensitive species following the increasing nutrient enrichment (Olsson et al. 2012; Snickars et al. 2015; Bergström et al. 2016b; Olsson 2019). Species belonging to the family Cyprinidae generally gain from more eutrophic conditions, while piscivorous species, such as European perch (*Perca fluviatilis*) and Northern pike (*Esox lucius*), require less nutrient-enriched waters. This is as also reflected in current standards for environmental assessment in the Baltic Sea region, where the abundances of perch and cyprinids, respectively, are key indicators (HELCOM 2018b). Since species vary in functional traits, changes in species composition are also attributed to effects on coastal food web processes and ecosystem services, for example through changes in the role of piscivorous species (Östman et al. 2016; Sundblad et al. 2020). However, fish are also influenced by several other factors, of which climate-related variables and fishing have particular roles, and the effects of different factors are often difficult to disentangle (Olsson et al. 2012; Snickars et al. 2015; Östman et al. 2017; Bergström et al. 2019; Bossier et al. 2021).

Björnöfjärden at the coast of the central Baltic Sea is a small (1.5 km^2^) enclosed bay that previously had bad eutrophication status, and which became subject to a pilot project for nutrient reduction measures in the 2010’s (Rydin et al. 2017; Rydin and Kumblad 2019). In fact, the Björnöfjärden project demonstrated the first full-scale eutrophication remediation in a coastal environment, including a geo-engineering method (injection of aluminium chloride into surface sediments to prevent phosphorus leakage to the water column), which led to a quick recovery and positive reactions in both the seabed and water column (Rydin et al. 2017). After the first three years, aluminium treatment was associated with about halved concentrations of total phosphorus and chlorophyll-*a*, and a halted formation of hydrogen sulfide above soft sediments at intermediate depths. Among ecological effects, an expanded depth range of small, attached algae was observed, attributed to improved water clarity, and initial increases in benthic macrofauna and fish were attributed to improved oxygen conditions (Rydin et al. 2017). The restored bay has been continuously monitored since the measures were started, enabling a unique opportunity to also evaluate long-term effects.

In this study, we evaluate the outcome of the Björnöfjärden coastal restoration project with a focus on changes in its fish assemblage over a time period of nearly one decade. The study is expected to strengthen understanding of how nutrient reduction measures may affect fish communities, and support the design of future efforts to restore coastal ecosystems. We particularly address how persistent any observed changes in the fish assemblage are over time, if changes can be attributed to the implemented restoration measures, and how the development of coastal fish relates to variables reflecting eutrophication status. Based on results from previous studies (as cited above), we hypothesize that the relative abundance of cyprinids will decrease with increasing water clarity, while the large predatory freshwater fishes perch and pike, and the marine herring (*Clupea harengus membras*), may increase with water clarity and improved oxygen conditions, overall leading to an increase in diversity and trophic level of the fish community.

## Materials and methods

### Study area and applied restoration measures

**Fig. 1:**
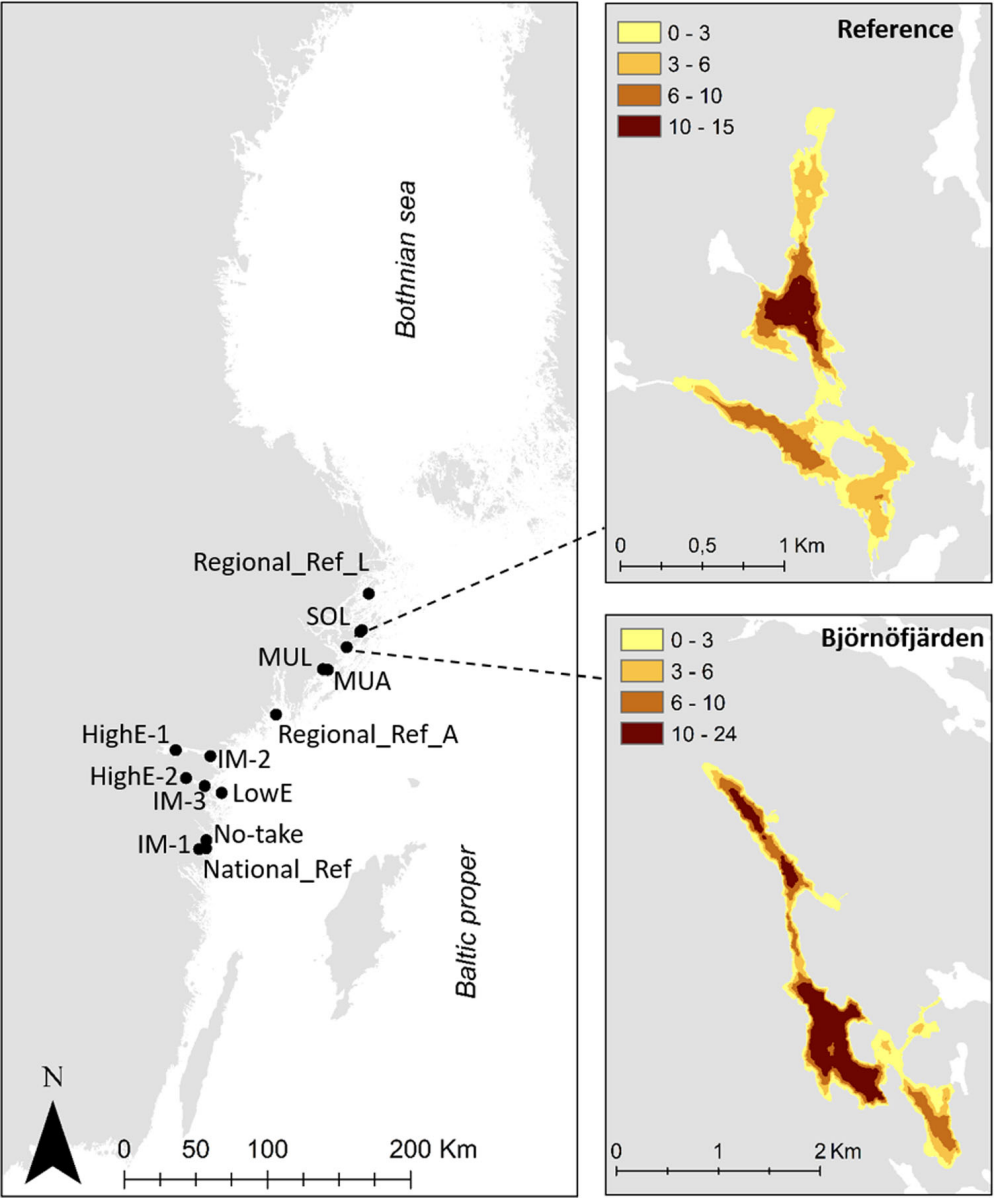
Location of the Björnöfjärden area in the northern Baltic Proper, and of the reference site for follow-up surveys. Colour symbols in the smaller maps indicate depth strata (m). The left map also shows the position of additional reference sites referred to in the present study (for details, see Fig. S1)

Björnöfjärden is located in the Stockholm archipelago on the coast of the central Baltic Sea. It is a small (1.5 km^2^) semi-enclosed basin with mean depth of 7 m and a maximum depth of 24 m (Fig. 1). Its drainage area contains agricultural fields, areas for horse keeping, rural residences, a tourism/conference facility, and forest. The bay has three relatively pronounced sub-basins, which stratify over summer. Hence, nutrient inputs accumulate easily, giving rise to hypoxic and anoxic conditions close to the seabed and in water masses below the thermocline (at around 6 m depth). Above the thermocline, the water is relatively clear during summer. Algal blooms occur mainly in spring and autumn in connection to vertical mixing.

Nutrient reduction measures were initiated in August 2012 and again in August 2013. During these occasions, aluminium chloride (Schütz et al. 2017) was injected to anoxic sediments to increase phosphorus retention. In parallel, land-based measures were implemented to reduce local inputs of nutrients from the drainage area (Rydin et al. 2017). Fishing was continuously allowed during most seasons. However, a seasonal closure was implemented from 2014 onwards, with a general fishing prohibition during 1 April-15 June, corresponding to the peak spawning season of coastal fish.

Monitoring to follow up on the measures was carried out in Björnöfjärden and in a reference site from year 2011 onwards, i.e., starting one year before the measures. The reference was selected to have similar trophic and topographical properties as Björnöfjärden, and geographical closeness. The reference was located 14 km north of Björnöfjärden, with a surface area of 0.7 km^2^, and maximum/mean depth 15/4 m (Fig. 1). The mean summer surface salinity (July and August) was 4.1–5.8 [psu] in Björnöfjärden and 3.7–5.3 in the reference site during years 2011–2020. No fishing restrictions were implemented in the reference.

Before the measures, the mean Secchi depth in summer was 4.2 m and the chlorophyll-*a* concentration 4.9 µg L^−1^ in Björnöfjärden, improving to 5.5 m and 3.0 µg L^−1^ directly following the aluminium treatment (Rydin et al. 2017). In the seabed, hydrogen sulphide formation ceased after the measures, attributed to improved light conditions allowing photosynthesis (Rydin et al. 2017). In all, the restoration induced a shift from poor to moderate/high status over the first years, based on the definitions of ecological status according to the EU Water Framework Directive (EC, 2000), in contrast to nearby areas which did not improve during the same time (Rydin et al. 2017; Kumblad and Rydin 2018).

### Fish surveys

Surveys of the adult fish assemblage were carried out using Nordic coastal multi-mesh gillnets using standardized methodology (HELCOM 2020) in 2011–2020. The monofilament nets are composed of nine panels with mesh sizes 10–60 mm in a geometric series with common ratio 1.25. In total, the gear is 1.8 m deep and 45 m long (5 m per panel). Fishing took place in summer (July/August) when the targeted coastal resident species are typically active and recruits from the preceding year have grown to be fully catchable in the gear. The nets were set in late afternoon and lifted the next morning. The number of fish per species and 1-cm length group was recorded separately for each station. Catch per unit effort (CPUE) was estimated as numbers per station and fishing occasion. Weight per unit effort (WPUE) was calculated from individual lengths and empirically determined length–weight relationships as applied in the Swedish national coastal fish database (www.slu.se/kul). The sampling design was similar for both Björnöfjärden and the reference. In all, 29 stations were sampled in each of the bays each year (Fig. S1), with the exception of the first year (2011) when 25 stations were sampled (four stations in the 0–10 m depth interval were not sampled). Most stations covered the 0–10 m depth interval, but five stations in Björnöfjärden and four in the reference were located at 10–20 m. Available data enabled quantitative analyses of catches within the depth interval 0–10 m, but the 10–20 m stratum had only few sampling points and the fish occurrences were too infrequent to allow separate analyses of this stratum.

Both Björnöfjärden and the reference also have suitable recruitment habitats for coastal fish (Erlandsson et al. 2021). Fish reproduction was monitored in late summer each year, using standardized pressure-wave sampling targeting juvenile fish (Sundblad et al. 2014; Bergström et al. 2021). This active sampling method is non-destructive for other biota than fish, and obtains data on the abundance of small fish in heterogeneous shallow water habitats where other sampling methods are lacking (Snickars et al. 2007). The method captures all species with swimbladders within an approximately 5-m radius of the detonation, yielding representative abundances and length distributions of fish with approximately 2–15 cm length. At each station, a detonation of 10 g Nobel prime explosive was applied, after which paralysed and killed fish were collected using catch nets for floating fish and by snorkelling for sunken fish, for determination of species, abundances and individual lengths. In all, 35 stations at 0.7–5 m depth were sampled in each of Björnöfjärden and the reference from 2012 onwards. The same stations were revisited each year. The year before the restoration, 2011, 10 stations were sampled in Björnöfjärden and 11 in the reference, with positions not always identical to those in subsequent years. CPUE was estimated as numbers per station and year. Soft substrates dominated at the sampled stations, and all sampled stations had vegetation to at least some extent (mean coverage 78% ± 44 (mean ± SD) in Björnöfjärden and 84% ± 44 in the reference). The vegetation was mainly *Najas marina* and *Vaucheria* spp in both Björnöfjärden and the reference site, but Characeans, filamentous and perennial macroalgae (representing both marine and freshwater species), and several species of freshwater phanerogams were also present.

In addition, migrations of fish from outside areas during the peak spawning time for freshwater fish in spring were monitored in years 2012–2017 using trap nets placed in the inlet of Björnöfjärden. These data were only used descriptively due to the absence of reference data and short time series.

### Hydrochemical data

Extensive monitoring has been carried out in Björnöfjärden and Table 1 gives an overview of variables of relevance for the present study. Hydrochemical data were sampled every two weeks during the growth season and otherwise monthly, from autumn 2011 onwards. Vertical profiles of temperature, oxygen levels and salinity were taken at the deepest position of each sub-basin, using a combi instrument. Composite samples of the surface water (epilimnion) as defined based on the profiles (Blomqvist 2001) were then collected at 3–5 positions in each sub-basin. The water samples were analysed for various variables, out of which total phosphorus and turbidity were used here, using data from the sampling position corresponding as closely as possible to each of the fish survey stations. Additionally, temperature and oxygen data were selected to represent the corresponding depth, based on the vertical profiles.

**Table 1:** Overview of data used in the study. All parameters were sampled in Björnöfjärden and the reference site during 2011–2020

Parameter	Unit	Sampling method	Occasion	Data points per occasion
Adult fish	CPUE, WPUE	Nordic coastal multi-mesh gillnets	Jul–Aug	29 in each of Björnöfjärden and reference^a^
Young-of-the-year fish	CPUE	Low impact underwater detonations	Aug–Sep	35 in each of Björnöfjärden and reference^b^
Temperature	°C	Combi instrument for hydrochemical profile	Twice a month during May–Sep, otherwise monthly	3 in Björnöfjärden 2 in reference
Dissolved oxygen	mgL^−1^			
Total phosphorus	µgL^−1^	Water sample	Twice a month during May–Sep, otherwise monthly	11 in Björnöfjärden 7 in reference
Turbidity	FNU			

^a^Applies to years 2012–2020. During 2011, 24 stations per site were sampled

^b^11 per site during 2011

### Analyses

#### Analyses of species composition

Relative levels of spatial and temporal variability in species composition of fish assemblages in Björnöfjärden and the reference were evaluated using Principal Coordinates analyses (PCO). For a wider comparison, additional data from gill net surveys conducted in adjacent sites using the same methodology were also included (Fig. 1). This encompassed the data included in Bergström et al (2016a), who explored variability in species composition in relation to ambient prevailing eutrophication levels, and from three more areas for which data from several years of monitoring were available (for details, see Fig. S1). The PCOs were based on similarity matrices applying the Bray–Curtis similarity index on root-transformed data, to weigh down highly abundant species. The analyses were conducted in parallel for abundance (CPUE) and biomass (WPUE) data, using average values for each fishing site and year. The analyses were carried out in PRIMER 7.0.

Subsequently, univariate analyses focusing on the dominating species and species groups, as well as assemblage-level indicators were carried out. For gill-net data, the individually assessed taxa were perch, herring, and cyprinids, where the latter included all species belonging to the taxonomic family Cyprinidae. This mainly included roach, bream, white bream and bleak, but also crucian carp, rudd, and tench. For the juvenile data, the dominating taxa were perch, cyprinids and pike. Adult pike was not included in the univariate analyses, although it occurs in the system, as it has poor catchability in the applied gear and hence was too infrequent in the data to construct reliable statistical models. At assemblage level, providing one data point per site and year, changes in diversity were assessed by the Shannon index (H’, as implemented in PRIMER 7.0), while changes in trophic status were assessed by the Mean Trophic Level of the catch (Christensen 1998) based on information on trophic levels of individual fish species from FishBase (Froese and Pauly 2022), and by the proportion of piscivores, computed as the ratio of fish with a trophic level ≥ 4.0, all based on WPUE data. 

### Effects of the restoration measure

All univariate data series were initially assessed by the Mann–Kendall trend test (McLeod 2011) to investigate the presence of monotonous changes over time. The presence of any treatment effect of the restoration was further assessed by generalized linear models (GLM) testing for an interaction between the fixed factor “Site” and the continuous explanatory variable “Year” regarding temporal developments in Björnöfjärden and the reference site. In the gill net data series, temperature at the time of fishing (“Temp_0”) was additionally included as a covariate, as this may affect fish activity and thereby catches in passive gears like gillnets (Bergström et al. 2016a; Naddafi et al. 2022). The temperature information was derived from the vertical profiles (Table 1), selecting sampling occasions corresponding as closely as possible to the time of fishing. Since hydrochemical data were sampled twice a month during summer, this meant a mismatch with the fish surveys of maximally ten days, usually less than one week. The juvenile data were assessed without covariates, as the here applied sampling method is not dependent on fish activity level. The presence of time lags in the response variables was assessed using partial autocorrelation functions (Östman et al. 2017), but indicated no significant autocorrelation at *p* < 0.05. Depth, which is another known predictor of fish catches, was not included as an explanatory variable, since depth-stratification was part of the sampling design with the same visited stations each year. Instead, the GLM analyses of the complete data set for gill nets were supplemented with separate analyses for the upper (0–6 m) and the middle (6–10 m), to investigate potential changes in the depth distribution of fish over time. The lowest (10–20 m) depth stratum could not be assessed separately due to low sample sizes. Models were run assuming a negative binominal distribution, to adjust for potential overdispersion in count data, and type III sums of squares, using the *MASS* package in R (Venables and Ripley 2002). Model fits were verified by inspection of diagnostic plots of initial runs, using the *boot* package (Davison and Hinkley 1997; Canty and Ripley 2021).

Additionally, for variables displaying a significant interaction term in the GLMs, generalized additive models (GAM, *k* = 4) were carried out separately for each site, to assess during which years after the treatment catches were different from the situation before treatment. Hence, the GAMs included “Year” as factor, covariates as in the GLMs, and departed from year 2012, which was applied as the year of comparison as it represented the last year before treatment effects could potentially be anticipated in the fish data. Species and species groups were assessed in parallel for CPUE and WPUE data. Assemblage-level indicators, which were only available at the aggregated level with one data point per year and site, were not assessed by the GLMs nor GAMs.

### Relation to variables reflecting eutrophication status

Relations between the focal taxa and pre-identified eutrophication-related variables were assessed using general additive models (GAM). The GAMs were carried out separately for each taxon and site (Björnöfjärden and reference).

**Fig. 2:**
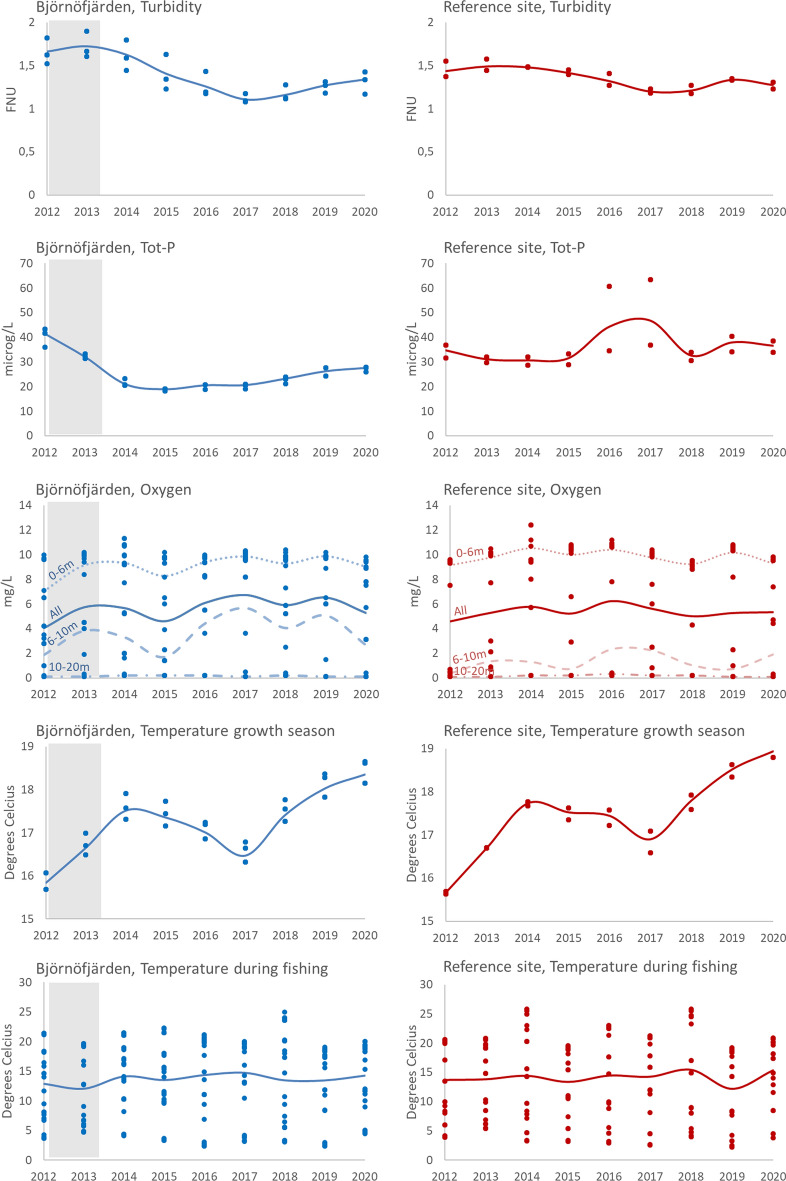
Environmental variables included in the GAMs for Björnöfjärden and the reference site. Grey areas show when the restoration took place in Björnöfjärden. Dots represent the data points used in the analyses for each year 2012–2020. Data were associated to stations as explained in the text (stations with identical values overlap in the graph). Lines show mean values, for illustrative purposes, for all data points. For oxygen data, mean values are also shown separately for each fished stratum. For more sampling details, see Table 1

For the gill-net data, variables representing turbidity, total phosphorus and oxygen levels were considered (Table 1, Fig. 2). For turbidity and phosphorus (“Turb” and “tot-P”), averages of mean monthly values during the preceding 24 months were used (Aug_*t-2*_ to July_*t-0*_), based on water samples from the position corresponding most closely to each fished station. A two-year lag was applied as these variables were assumed to mainly influence fish through effects on feeding conditions, and hence growth, over time (Sandström and Karås, 2002; Bergström et al. 2013). For year 2012, only data from 12 months back was available. Applied oxygen values represented the year of fishing, as oxygen conditions were assumed to affect the fish distribution through behaviour, with fish avoiding areas with low oxygen concentrations. Oxygen data were obtained from the hydrochemical profiles, using the data point corresponding most closely to the position, time and depth of each fished station. Further, a variable representing the average surface temperature during previous growth seasons (“Temp_Prev”) was included to account for potential effects of temperature on survival and growth of fish recruiting to the gear. This was calculated as the mean for months May to Sept during the preceding two years, and of months May–July during the year of sampling, using data from 0.5 m depth. Also, temperature during the time of fishing (“Temp_0”) was included as potential explanatory variables, calculated as described above (Fig. 2).

Analyses of the juvenile fish abundance data focused on turbidity and total phosphorus levels, as above. Information on oxygen levels was not relevant for the juvenile fish sampling, as the shallow areas where juveniles occur and were sampled (mainly at 0–3 m) are not affected by oxygen deficiency. The “Temp_Prev” variables was also included for the juvenile data, but computed as the mean surface temperature per sub-basin during the time between spawning and sampling (May-Aug) each year, to represent potential effects on the growth and survival of juveniles during their first year. In addition, the mean biomass of the focal species in the previous year´s gill net survey “Catch_prev”, was included as a covariate to assess the potential contribution of the adult population size on recruitment. This was calculated as the mean WPUE per sub-basin, and data were linked to the corresponding sampling positions for the juvenile survey. Possible collinearities among explanatory variables were checked based on Variance Inflation Factors (VIF). In cases when variables contributed to VIF > 3 (Zuur et al. 2010), parallel runs were conducted excluding and including these. This was the case for the oxygen variable (VIF < 5.1 in Björnöfjärden and < 4.7 in the reference when including, while < 1.7 and < 1.3, respectively, when excluding the oxygen variable).

The GAMs were performed assuming a negative binominal distribution, with three degrees of freedom (*k* = 4) to assess the primary type of relationships (linear, unimodal or higher) between response and explanatory variables. Model fits were verified by inspection of diagnostic plots. Final models were identified by backwards selection until only significant variables (*p* < 0.05) remained and AIC values minimized. The analyses were carried out in R using the *mgcv* package (Wood 2017).

## Results

### Species composition

**Fig. 3:**
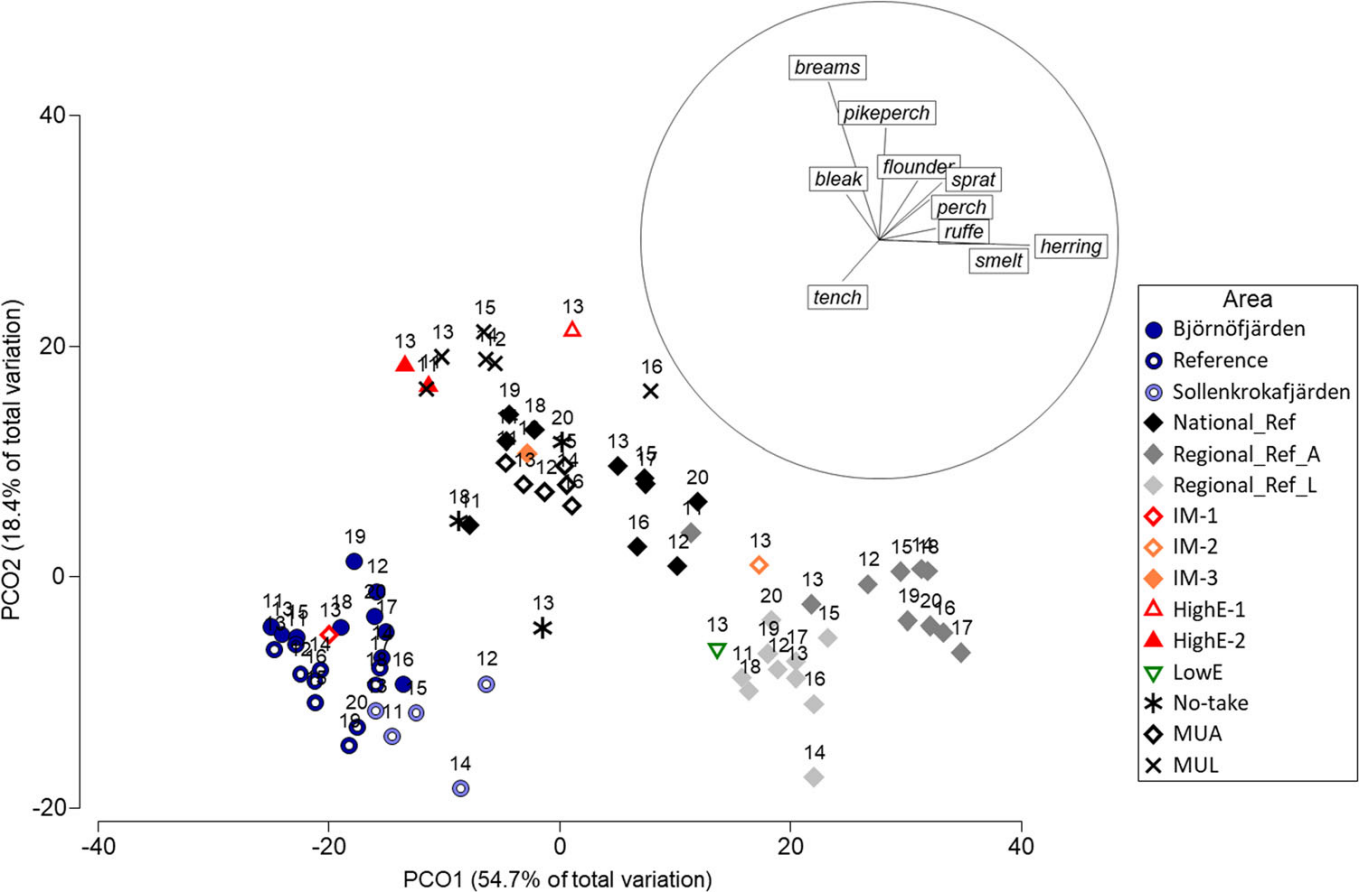
Multivariate biplot from PCO addressing similarities in species composition between sites in the study area (Björnöfjärden and reference site) and additional adjacent monitoring sites (see legend, Fig. 1 and Fig. S1 for more information). The PCO was based on Bray–Curtis similarities and biomass data from the gill-net survey. The inset vector plot illustrates the characteristic species for sites in different directions in the biplot. The vectors show species contributing most to the observed pattern based on a multiple correlation > 0.2, and the circle corresponds to a vector length = 1. Sites characterised by breams, pikeperch and flounders are mainly located in the upper end of the graph, while sites characterised by herring, smelt, perch and ruffe are located to the right end

Björnöfjärden and the reference site had high similarities in species compositions, as shown in the PCO where these sites are located close to each other in the sample biplot (Fig. 3). Björnöfjärden and the reference were mainly characterised by tench, bleak and breams, as illustrated by that vectors for these species point to the left along the first PCO axis, which represents the main part (54.7%) of the total variation. The characteristic species compositions of both sites were retained over all sampled years, as shown in the PCO by that data points representing the same site in different years were clustered closer together than data points representing different sites. Hence, no major shifts in species composition occurred over the studied time period.

In all, 20 taxa were recorded, out of which perch, roach, bleak, herring, and breams dominated (Table 2). Most fish species were of freshwater origin. Species of marine origin were herring, sprat and straightnose pipefish, with herring being the only species recorded more than occasionally. Several species typical for coastal areas in this part of the Baltic Sea were relatively poorly represented, which was also reflected in a relatively low Shannon index (H’); on average 2.17 (SD = 0.10) in Björnöfjärden and 2.01 (SD = 0.06) in the reference over the studied years, as compared to 2.39–2.44 in adjacent national and regional monitoring areas. The indices were similar to those of the adjacent Sollenkrokafjärden (H’ = 2.10) and two sites categorized as highly eutrophic in Bergström et al. (2016a, b) (H’ = 2.27 for HighE-1 and 2.03 for HighE-2). Several species observed to be migrating into Björnöfjärden in spring based on the trap net survey were however not observed in the gill net sampling in August (Table 2).

**Table 2:** Species registered in the different fish surveys. Values are % of abundance based on mean annual catch per unit effort over years 2012–2020 for gill net and juvenile fish surveys, and 2012–2017 for trap nets. F = freshwater origin, M = marine origin. * Belonging to family Cyprinidae. BJO = Björnöfjärden, REF = Reference. Trap net data were only collected from Björnöfjärden

Species		Gill nets		Juvenile fish survey		Trap nets
Scientific name	English name	BJO	REF	BJO	REF	BJO
*Abramis brama/Blicca bjoerkna*^a^	Bream/white bream (F)*	18.2	4.8	1.9	2.1	
*Abramis brama*	Bream (F)*	–	–	–	–	3.2
*Alburnus alburnus*	Bleak (F)*	13.4	5.4	5.9	2.4	0
*Anguilla anguilla*	Eel (M)	0	0	0	0	0.3
*Blicca bjoerkna*	White bream (F)*	–	–	–	–	3.5
*Carassius carassius*	Crucian carp (F)*	0.2	0	0.1	0.1	0.1
*Clupea harengus*	Herring (M)	3.5	0.8	0	0	2.3
*Coregonus maraena*	Whitefish (F)	0	0	0	0	0
*Esox lucius*	Pike (F)	0.2	0.2	1.2	2.7	0.3
*Gasterosteus aculeatus*	Three-spined stickleback (M)	0	0	0.2	0	0
*Gobius niger*	Black goby (M)	0	0	0.3	0.2	0
*Gymnocephalus cernua*	Ruffe (F)	1.6	2.7	0.2	0.9	2.8
*Leuciscus idus*	Ide (F)	0	0	0.1	0	0
*Leuciscus cephalus*	European chub (F)*	0	0	0	0	0
*Lota lota*	Burbot (F)	0	0	0	0	0
*Myoxocephalus quadricornis*	Fourhorn sculpin (F)	0	0	0	0	0
*Onchorhynchus mykiss*	Rainbow trout	0	0	0	0	0
*Osmerus eperlanus*	Smelt (F)	0.2	0	0	3.6	0
*Perca fluviatilis*	Perch (F)	36	42	15	24	34
*Platichthys flesus*	Flounder (M)	0	0	0	0	0
*Pomatoschistus microps/P. minutus*^*1*^	Common goby/sand goby (M)	0	0	1.4	3.2	0
*Pungitius pungitius*	Nine-spined stickleback (M)	0	0	0	0	0
*Rutilus rutilus*	Roach (F)*	26	37	72	59	51
*Salmo trutta*	Trout (N/A)	0	0	0	0	0
*Scardinius erythrophthalmus*	Rudd (F)*	0.8	0.8	0	0	0.7
*Sprattus sprattus*	Sprat (M)	0.2	0	0	0	0
*Sander lucioperca*	Pikeperch (F)	0	0	0	0	0
*Tinca tinca*	Tench (F)*	0.8	1.3	0.2	0.1	1.3
*Vimba vimba*	Vimba bream (F)*	0	0	0	0	0

^a^Not distinguished due to high morphological similarity

### Changes over time in individual taxa and assemblage-level indicators

**Fig. 4:**
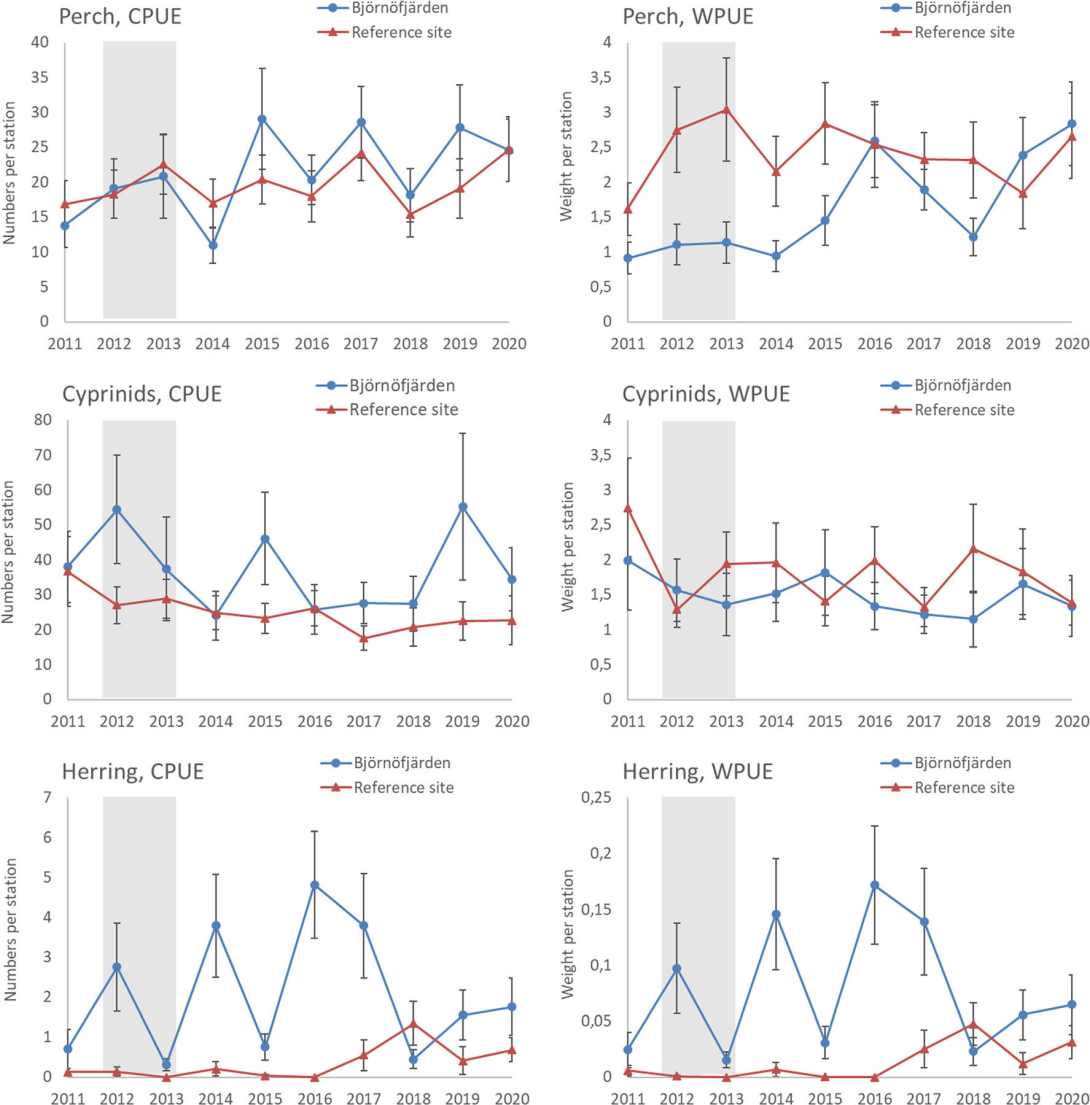
Development over time of perch, cyprinids and herring in Björnöfjärden and the reference site, based on the gill net surveys. Grey areas show when the restoration took place in Björnöfjärden. Catches per unit effort are shown based on abundances (CPUE) and biomass (WPUE) data. Error bars show standard error

**Fig. 5:**
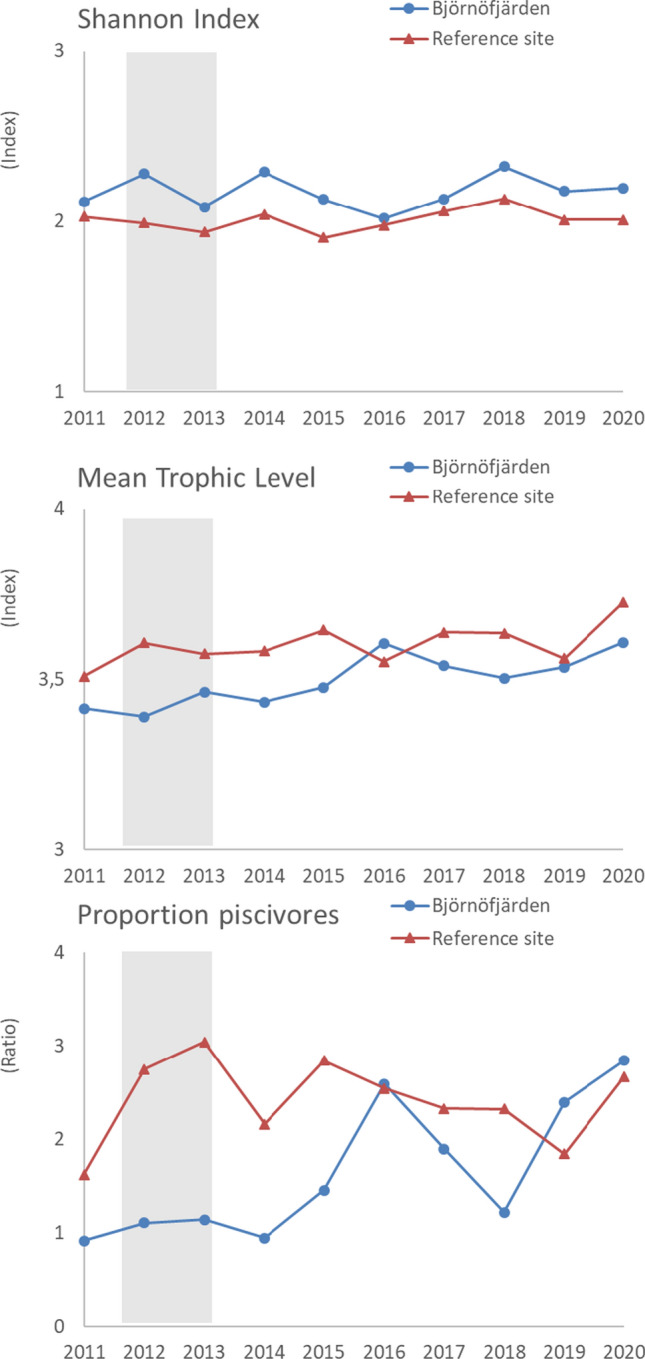
Development over time in the assemblage-level indicators in Björnöfjärden and the reference site. Grey areas show when the restoration took place in Björnöfjärden

Initial screening of the data showed that the WPUE of perch in Björnöfjärden increased monotonously over 2011–2020 (Mann Kendall, tau = 0.644, *p* = 0.012), while no changes were observed in the other focal variables of the gill net data (*p* > 0.05). Cyprinids decreased initially after the treatment in Björnöfjärden (Fig. 4), but the presence of high catches at the end of the time series interrupted any longer-term trend. The increase was particularly evident in 2019, where inspection of raw data revealed high abundances of small-sized breams at one of the stations. In addition, a decreasing trend occurred in cyprinid CPUE in the reference site (tau = − 0.600, *p* = 0.020), which was attributed to high values in the first year of study. Catches of herring were clearly lower than the catches of perch and cyprinids and showed strong variability between years. At the assemblage level, there were no monotonous changes over time in the Shannon index. However, the Mean Trophic Level and the proportion of piscivores increased in Björnöfjärden (tau = 0.689, *p* < 0.01 in both series), while showing no temporal trend in the reference site (*p* = 0.21 and 0.37, for MTL and PP, respectively; Fig. 5).

**Fig. 6:**
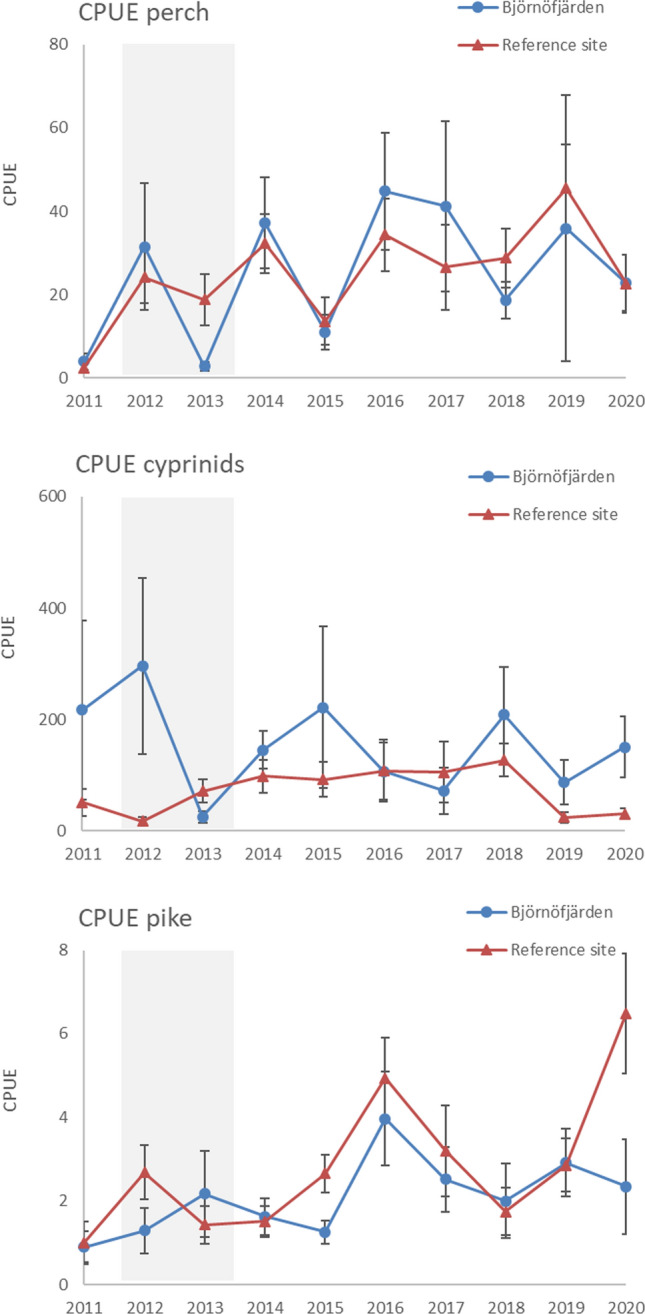
Development over time in the abundance of juveniles of perch, cyprinids and pike in Björnöfjärden and the reference site. Error bars show standard error. Grey areas show when the restoration took place in Björnöfjärden

There were no monotonous trends in the juvenile data for perch nor cyprinids (Mann–Kendall test, *p* > 0.1). Juvenile pike showed an increasing trend in the reference site (tau = 0.556, *p* = 0.318), but not in Björnöfjärden (tau = 0.467, *p* = 0.073; Fig. 6).

### Assessment of treatment effects

Effects of the nutrient reduction treatment were assessed including temperature at the time of fishing (Temp_0) as a covariate. The GLMs showed a clear positive effect of temperature at the time of fishing (‘Temp0’) in the total data set for all taxa, mainly attributed to catches in the intermediate (6–10 m) and not the shallowest (0–6 m) depth interval (Table 3).

**Table 3:** Output of GLMs testing for differences in the temporal development of fish variables in Björnöfjärden and the reference site. Analyses were performed over years 2011–2020 for perch, cyprinids and herring in the gill net survey (CPUE and WPUE) and for perch, cyprinids and pike in the juvenile fish survey (CPUE). Temperature at the time of fishing, “Temp0”, was included as a covariate for the gill net data. A significant interaction “Site*Year” indicates a different directional development and a potential effect of the restoration (model output for individual factors not shown). Values show F. df = degrees of freedom. Significance of variables: *** for p < 0.001, ** for p < 0.01, * for p > 0.05. ns = not significant

Taxon	Depth	df	WPUE		CPUE	
			Temp0	Site*Year	Temp0	Site*Year
Gill net data						
* Perch*	All (0–20)	1569	342***	18.8***	288***	9.9**
	0–6 m	1288	(ns)	11.6***	(ns)	(ns)
	6–10 m	1190	45.8***	5.4*	24.9***	6.9**
* Cyprinids*	All (0–20)	1569	281***	(ns)	235***	10.6**
	0–6 m	1288	(ns)	(ns)	(ns)	(ns)
	6–10 m	1190	114***	(ns)	62.1***	(ns)
* Herring*	All (0–20)	1569	6.9**	8.3**	16.2***	13.6***
	0–6 m	1288	(ns)	5.3*	(ns)	5.9*
	6–10 m	1190	6.4*	5.3*	10.4**	13.6***
Juvenile survey						
* Perch*	0–5 m	1650	–	–	–	(ns)
* Cyprinids*	0–5 m	1650	–	–	–	(ns)
* Pike*	0–5 m	1650	–	–	–	(ns)

A potential treatment effect was observed for perch and herring, based on an interaction between ‘site’ and ‘year’ (*p* < 0.01) in GLMs indicating different temporal developments between Björnöfjärden and the reference. Inspection of interaction effects plots confirmed an increasing trend for perch in Björnöfjärden, and not in the reference site. Subsequent GAMs to further characterize the temporal variability indicated particularly low perch CPUE in year 2014 (*p* < 0.01) and high CPUE in 2018 (*p* < 0.05), as well as high WPUE in years 2016 (*p* < 0.01) and 2020 (*p* < 0.05), as compared to year 2012 that represented the last year before the treatment was implemented. Herring CPUE and WPUE increased slightly over time in both sites, however with a steeper increase in the reference site. The subsequent GAMs identified a peak in herring CPUE in Björnöfjärden in the middle of the time series (*p* < 0.01), and in the reference in 2018 (*p* < 0.05). The results were similar for the separately assessed depth intervals 0–6 m and 6–10 m, respectively (Table 3).

Cyprinid WPUE showed no treatment effect. For cyprinid CPUE, a significant interaction mainly reflected high values in the reference site during the very first sampling year, and the same result was no longer observed (*p* = 0.93) when the GLM was re-run excluding the first year. GAMs addressing the temporal development of Cyprinid CPUE identified particularly low catches in year 2016 in the reference site, compared to the year 2012 (*p* < 0.01). For Björnöfjärden, lower Cyprinid CPUE compared to year 2012 were seen in most but not all subsequent years, namely 2014, 2016–2018 and 2020 (*p* < 0.01; Table 3).

The GLMs on juvenile data showed no interactions between ‘site’ and ‘year’, (*p* = 0.841 for perch, *p* = 0.312 for cyprinids, *p* = 0.476 for pike). An increasing trend for pike was noted in both sites, while no changes over time were seen in perch or cyprinids (Table 3).

### Effects of environmental variables

The analyses of relationships to environmental data confirmed strong relationships with temperature during fishing for the gill net data (*p* < 0.001, Table 4). Catches of perch and cyprinids generally increased up until temperatures of around 18–20 °C, after which they levelled off or decreased. They showed no relationships to temperature in the previous growth season, with the exception of perch CPUE in the reference area where data indicated a negative relationship (*p* < 0.01).

**Table 4:** Summary of results from GAMs to test for relationships between the focal taxa and environmental variables. Analyses were carried out separately for Björnöfjärden (BJO) and the reference site (REF), for data on abundance (CPUE) and biomass (WPUE) per unit effort. Values denote Chi^2^. For significant variables (p > 0.01), the direction of response is indicated as interpreted from partial response plots: +  = positive,—= negative, * = unimodal. Double symbols denote p < 0.001. (ns) = not significant. NA = not applied. R^2^ = Adjusted R^2^, D^2^ = Deviance explained

Response variable		Explanatory variables					Model statistics	
Taxon SITE	Unit	Temp. during fishing	Temp. growth season	Oxygen	Turbidity	Tot-P	*R*^2^	*D*^2^
Perch BJO	CPUE	336 (++)	(ns)	(ns)	(ns)	(ns)	0.428	63.1
	WPUE	60.3 (++)	(ns)	9.8 (**)	(ns)	(ns)	0.396	52.3
Perch REF	CPUE	92.2 (++)	8.8 (−−)	14.2 (+ +)	11.9 (−−)	(ns)	0.554	62.1
	WPUE	61.3 (++)	(ns)	(ns)	(ns)	(ns)	0.435	58.0
Cyprinids BJO	CPUE	180 (++)	(ns)	15.7 (−−)	3.9 ( +)	(ns)	0.309	62.6
	WPUE	70.7 (++)	(ns)	(ns)	11.1 (+ +)	5.5 (−−)	0.402	58.1
Cyprinids REF	CPUE	425 (++)	(ns)	(ns)	14.9 (+ +)	(ns)	0.374	58.7
	WPUE	123 (++)	(ns)	(ns)	(ns)	(ns)	0.348	58.4
Herring BJO	CPUE	65.8 (**)	3.5 (–)	(ns)	(ns)	(ns)	0.339	35.1
	WPUE	19.2 (**)	(ns)	(ns)	(ns)	(ns)	0.346	31.1
Herring REF	CPUE	NA	NA	NA	NA	NA	0.010	15.5
	WPUE	NA	NA	NA	NA	NA	< 0.01	< 1.0

Regarding variables related to eutrophication, catches of perch showed no relationships to turbidity nor total phosphorus in Björnöfjärden, while perch CPUE showed a negative relationship with turbidity in the reference site (*p* < 0.001, Table 4). For oxygen, there was a positive relationship with CPUE in the reference site, and a unimodal relationship to perch WPUE in Björnöfjärden, indicating highest catches at oxygen levels around 6 mgL^−1^. The GAMs were run with and without oxygen as an explanatory variable, to control for potential effects of its collinearity with temperature during fishing. Inclusion of oxygen as an additional variable increased the deviances explained only marginally. Results for the other variables were the same in GAMs run with and without the oxygen variable.

Catches of cyprinids showed a positive relationship to turbidity in Björnöfjärden, as well as in the reference site for the CPUE data (Table 4). In addition, there was a negative relationship between Cyprinid WPUE and total phosphorus in Björnöfjärden (*p* < 0.01). Cyprinid CPUE in Björnöfjärden showed a negative relationship with oxygen. When the GAMs were re-run omitting oxygen, the results for the other variables remained and the relationship between Cyprinid CPUE and turbidity enforced to *p* < 0.001.

GAMs for herring in the gill nets yielded low deviances explained, but were to some extent interpretable for the Björnöfjärden data, indicating highest catches at temperatures around 10–13 °C, a negative relationship to temperature in the previous growth season for herring CPUE, and no relationship to the other variables (Table 4).

GAMs for the juvenile sampling data yielded poor deviances explained (< 8.1% in all cases), and were not interpreted further.

## Discussion

Following the nutrient abatement, Björnöfjärden has been one of few water bodies at the Swedish Central Baltic Sea coast meeting or being close to meeting the criteria for good ecological status as set by the EU Water Framework Directive (EC 2000; Walve and Rolff 2021). In the present study, we investigated how the implemented measures affected the temporal development of coastal fish. Hence, an important novelty of the study was that it evaluated responses of coastal fish to nutrient reductions in a controlled setting, while previous studies have focused on responses to increasing ambient nutrient concentrations, or been conducted in spatial eutrophication gradients (Snickars et al. 2015; Bergström et al. 2016a, 2019; Olsson 2019; Sundblad et al. 2020). This enabled an assessment of fish species and community responses in relation to systematically monitored water chemistry data, while previous studies have been carried out with much coarser data, or using proxies for eutrophication status, such as water transparency.

The results indicated some positive responses for coastal fish after restoration, although there were no conclusive patterns over the full ten-year time period. Comparisons between Björnöfjärden and the unrestored reference site indicated a positive effect of nutrient reduction on perch, while cyprinid abundances were lowered in most years after the treatment. These changes were also reflected in increases over time in the mean trophic level and proportion of piscivores in the fish assemblage. The study did not show any indications of local fish communities taking harm from the aluminium treatment. The observed decrease in cyprinid abundance was likely a response to changes in turbidity levels following the treatment, although this effect was not persistent over longer time. The analyses in relation to environmental variables showed that cyprinid abundance changed in relation to changes in ambient turbidity also in the untreated reference.

The observations from Björnöfjärden are in line with previous studies in eutrophication gradients (Snickars et al. 2015; Bergström et al. 2016a, 2019; Olsson 2019; Sundblad et al. 2020), supporting that the observed changes in fish communities are linked to improved eutrophication status. In an aluminium-treated eutrophied lake in Denmark, increases in perch and declines in roach (*Rutilus rutilus*) were observed, together with a shift in species distribution towards deeper areas following improved oxygen conditions (Lund et al. 2010). In that study, the shifts were, however, pronounced and quick, likely reflecting a stronger treatment effect with a several-fold decrease in phosphorus concentrations and almost a doubling in water transparency. We have not found other studies that are directly comparable, as most restoration studies available have involved combinations of biomanipulations (usually the removal of cyprinids) with treatments to decrease nutrient loading (Jeppesen et al. 2012).

The rather weak responses observed here were likely attributed to eutrophication levels fluctuating over the duration of the survey, in combination with a slow response time in the relatively long-lived fish species. Phosphorus levels first decreased in connection to the aluminium treatment in the restored area, but then increased from the year 2016, although remaining at lower levels than prior to treatment (Fig. 2). This was partially attributed to a series of breakdowns in a wastewater system on the western shore leading to temporarily enhanced local loading. In addition, nutrient reduction measures in a water body can in the long term generally not be expected to achieve more benign conditions than in the water bodies to which it is connected, unless the measures are regularly repeated (Bryhn et al. 2017). In Nämdöfjärden, the area outside of and connected to Björnöfjärden, years with elevated phosphorus levels were also observed during the study period, attributed to concurrent shifts in nutrient levels in the open Baltic Sea (Walve and Rolff 2021).

The more detailed analyses in relation to environmental variables, however, confirmed a positive relationship between cyprinid abundance and eutrophication, as indicated by mean turbidity levels during the preceding 24 months. Perch abundance was not related to turbidity in Björnöfjärden, although a negative relationship in the reference site could possibly support a response also for this species. Regarding oxygen levels, cyprinid catches appeared to decrease and perch increase with increasing oxygen, although these results were difficult to link to likely mechanisms and might rather reflect correlations between oxygen levels and other factors, such as depth. Similarly, a negative relationship between cyprinid biomass and phosphorus levels in the restored site appeared spurious. For herring, the inconclusive results likely reflected overall low catches and that populations have a wide distribution and are therefore largely determined by processes outside the study area. Potentially, the strong interannual variability in herring might reflect responses to years with better oxygen conditions. However, oscillating patterns could also occur for other reasons, such as variable recruitment or density-dependent interactions between cohorts leading to competition or cannibalism (Ricard et al. 2016).

No treatment effects were observed in the juvenile data. Here, the possibility to detect changes over time was hampered by an insufficiently low sampling effort in relation to the large spatial variability in distributions. Previous studies on juvenile fish have shown strong effects of turbidity on the spatial distribution of especially percids and cyprinids in Baltic Sea coastal waters (Sandström and Karås, 2002; Bergström et al. 2013; Erlandsson et al. 2021).

The fact that the studied coastal fish communities were clearly dominated by species of freshwater origin, which are typically local with limited dispersal, suggests a high probability that the fish targeted in the summer gill net surveys are actually present in the bays during their full life cycles. Still, a significant proportion of fish hatched in Björnöfjärden likely perform feeding migration outside of the bay, which could contribute to explaining variability among years. The studies of fish migrations to Björnöfjärden carried out in spring confirmed that several species perform spawning migrations to and from the bay, but also revealed that several species prevailing in the areas in spring were not captured in the late summer gill net surveys. Poor oxygen conditions in deeper parts of the bay could decrease incentives for fish to stay in the bay during summer, through direct avoidance or as the result of lowered availability of benthic prey species.

Both Björnöfjärden and the reference had a relatively simple species composition compared to nearby reference sites for environmental monitoring, with no changes observed over time. The lower oxygen conditions in combination with the fact that the bays are relatively enclosed could explain the exclusion of some species which might otherwise be expected, such as flounder (*Platichthys* spp) and cod (*Gadus morhua*). Besides reaching the environmental objectives for water quality, one major goal with the nutrient reduction measures was to reduce the production and subsequent settling of organic matter enough to maintain oxic hypolimnic conditions also during summer stagnation. Achieving this was foreseen to facilitate the binding of phosphorus to iron in the sediment also after the added aluminium has been saturated with phosphorus (Rydin and Kumblad 2019), hence interrupting the “vicious circle” which arises when the turnover of phosphorus increases the more hypoxia expands, hence even further amplifying primary production and oxygen demand (Vahtera et al. 2007). In addition, maintaining oxic conditions below the thermocline throughout summer would open this habitat for fish species dependent on cold water during summer. A few years after the treatment, there were signs of oxygen recovery below the thermocline (Rydin et al. 2017). However, a major shift in the redox environment has not yet been observed. The load of nutrients and degradable organic matter might still be too large to regain the deeper habitats of the bay, which in turn may prevent any significant shifts in fish community composition.

Although the study focused on effects of nutrient reduction, other factors might also have affected the results with regards to fish, most notably the simultaneously implemented seasonal spawning closure but also the creation of a small (0.63 ha) wetland. Any potential effects of these were not possible to disentangle. Unfortunately, there is no information of the levels of fishing pressure in the studied sites, which are mainly subject to recreational fishing. Recent studies in the same coastal region have demonstrated no responses of perch nor roach (the most abundant cyprinid) to seasonal spawning closures (Eklöf et al. 2023), as well as wetlands (Hansen et al. 2020). These measures could, however, have benefited pike (Nilsson et al. 2014; Hansen et al. 2020), potentially leading to cascading effects on cyprinids. Pike is a key piscivore in the area, but was not addressed in the current study due to low catchability in the applied gears. Pikeperch (*Sander lucioperca*) is another predatory fish in Baltic Sea coastal areas that is impacted by fishing and hence could be benefitted by fishing regulations (Bergström et al. 2019). However, pikeperch was not present in the study area, which could be expected as it typically resides in areas with an even lower water transparency, below 2 m (Bergström et al. 2013).

Notably, the overall responses of fish in the current study were clearly less pronounced than what has been observed for year-round fishing closures, where fast recovery times, especially of large predatory fish, and food web effects similar to those under nutrient reductions due to a restored top-down control (Östman et al. 2016; Bergström et al. 2019, 2022). The results, hence, suggest that for a quick restoration of fish communities from eutrophication-induced degradation, a combination of nutrient reduction measures with full fishing closures would be preferred.

## Conclusions

- Eutrophication reduction gave slow responses in fish communities, as compared to alternative measures to restore fish, such as fishing closures.
- Mean trophic level increased over time in the restored area, attributed to an increase in perch biomass and an initial, but subsequently interrupted, decrease in cyprinids.
- The weak responses were likely partially attributed to an attenuation of the effect of the nutrient reduction over time, as full recovery of the deeper benthic habitats was not attained, and to continued external loading.
- The temporal development of cyprinids responded to decreasing turbidity levels both in the restored area and the reference site.

### Supplementary Information

Below is the link to the electronic supplementary material.

Supplementary file1 (PDF 326 KB)
