# Supplementary material for: Fish community responses to restoration of a eutrophic coastal bay
Source: Ambio. 2023 Aug 5;53(1):109–25. doi: 10.1007/s13280-023-01907-3 (PMC10692049; doi:10.1007/s13280-023-01907-3)
Supplement: Supplementary file 1 — Supplementary file1 (PDF 326 KB) [file 13280_2023_1907_MOESM1_ESM.pdf]

Ambio

Supplementary Information

This supplementary information has not been peer reviewed.

Title**: Fish community responses to restoration of a eutrophic coastal bay**

Figure S1: Positions for sampled stations in Björnöfjärden and the reference: a-b) Gillnet fishing stations. c-d) Juvenile surveys. e-f) Hydrochemical monitoring. For details, see Table 1.

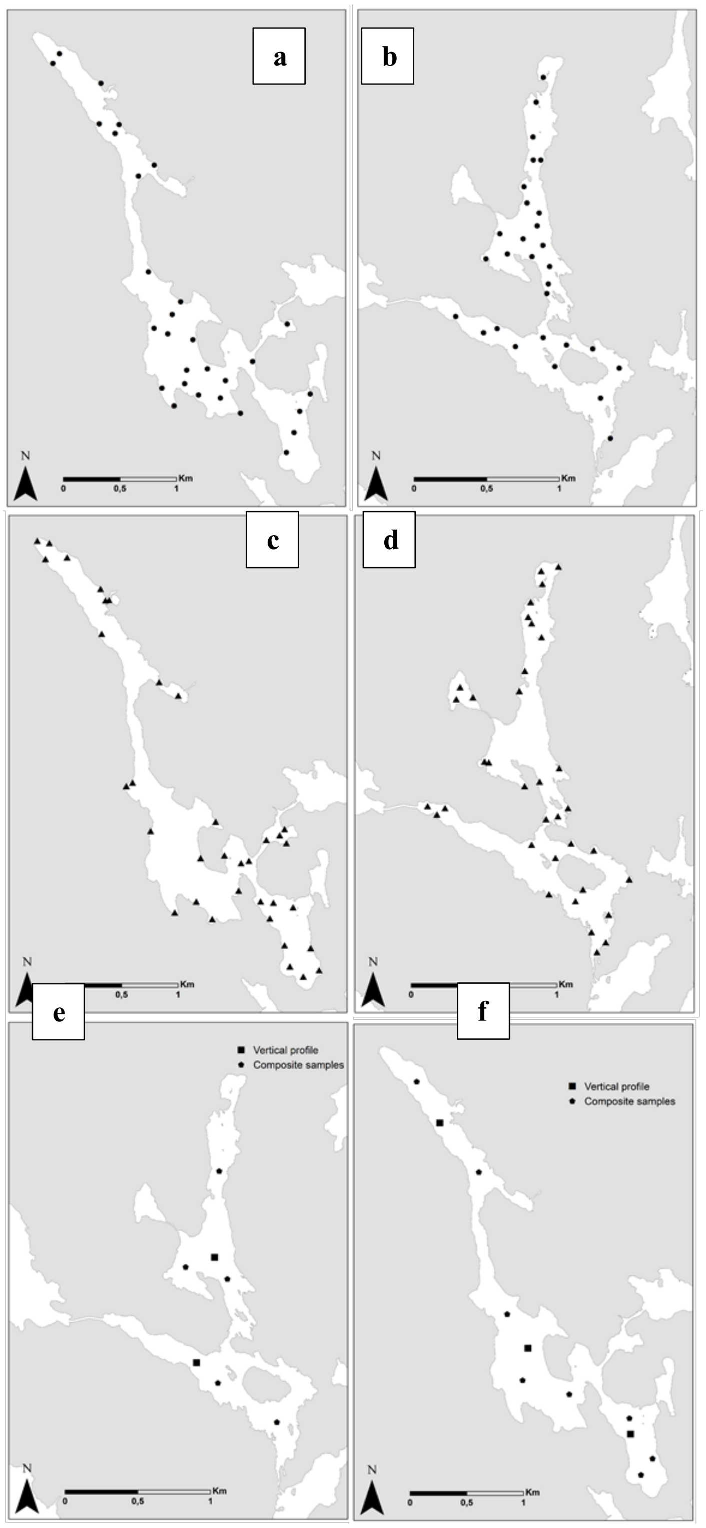

Table S2. Full names of the survey areas included in Fig, 1, with purpose and key characteristics. Data downloadable at (www.slu.se/kul).

**Short name**	**Full name**	**Purpose. Source**	**Years included**
Björnöfjärden	Björnöfjärden	Follow up on restoration measure (this study)	2011-2020
Reference site	Älgöfjärden-Fjällsviksviken	Reference site in this study (not restored)	2011-2020
National_Ref	Kvädöfjärden	National coastal fish monitoring	2011-2020
Regional_Ref_L	Lagnö	Regional coastal fish monitoring	2011-2020
Regional_Ref_A	Asköfjärden	as above	2011-2020
IM-1	Kaggebofjärden	Bergström et al. 2016a	2013
IM-2	Bråvikens kustvatten - Ost Lönö	as above	2013
IM-3	Trännöfjärden	as above	2013
HighE-1	Inre Bråviken	as above	2013
HighE-2	Inre Slätbaken	as above	2011.2013
LowE	Kärrfjärden	as above	2013
No-take	Licknevarpefjärden	as above. fishing closure	2013.2018
MUL	Muskö-Lännåkersviken	Follow up on fishing regulations – no take area	2011-2016
MUA	Muskö-Askviken	Reference site to Muskö-Lännåkersviken	2011-2016
SOL	Sollenkrokafjärden	Tentative reference to Björnöfjärden (closed 2016)	2011-2015
